# Beneficial Regulation of Cellular Oxidative Stress Effects, and Expression of Inflammatory, Angiogenic, and the Extracellular Matrix Remodeling Proteins by 1α,25-Dihydroxyvitamin D3 in a Melanoma Cell Line

**DOI:** 10.3390/molecules25051164

**Published:** 2020-03-05

**Authors:** Neena Philips, Philips Samuel, Thomas Keller, Asma Alharbi, Samar Alshalan, Sara-Ali Shamlan

**Affiliations:** 1 Professor of Biology, Fairleigh Dickinson University, H-DH4-03; 1000 River Road, Teaneck, NJ 07666, USA; 2 Department of Biological Sciences, Fairleigh Dickinson University, Teaneck, NJ 07601, USA; philipssamuel118@gmail.com (P.S.); bluemax00@aol.com (T.K.); Asma536@student.fdu.edu (A.A.); Samar12@student.fdu.edu (S.A.); Sara_25@student.fdu.edu (S.-A.S.)

**Keywords:** oxidative DNA/RNA damage, superoxide dismutase, p53, interleukin-1, tumor necrosis factor-α, transforming growth factor-β, vascular endothelial growth factor, matrix metalloproteinases

## Abstract

The causes of cancer include the cellular accumulation reactive oxygen species (ROS), which overrides the cellular antioxidants such as superoxide dismutase, from intrinsic aging, genetics, and exposure to environmental pollutants and ultraviolet (UV) radiation. The ROS damage biomolecules such as DNA (including p53 gene), RNA, and lipids, and activate inflammatory, angiogenic, and extracellular matrix (ECM) remodeling proteins; which collectively facilitate carcinogenesis. The 1α,25-dihydroxyvitamin D3 (Vitamin D) has anti-carcinogenic potential from its antioxidant, anti-inflammatory, and endocrine properties. We examined the anti-carcinogenic mechanism of vitamin D through the beneficial regulation of oxidative stress effects (oxidative DNA/RNA damage, superoxide dismutase expression, membrane damage, and p53 promoter activity), and expression (at the protein, mRNA and/or promoter levels) of inflammatory mediators (interleukin-1 (IL-1) and tumor necrosis factor-α (TNF-α)), angiogenic mediators (transforming growth factor-β (TGF-β), and vascular endothelial growth factor (VEGF)), and the ECM remodeling proteins (matrix metalloproteinases (MMP)-1 and MMP-2) by vitamin D in melanoma cells. Vitamin D inhibited oxidative DNA/RNA damage and membrane damage; and stimulated superoxide dismutase expression and p53 promoter activity in melanoma cells. It inhibited the expression of IL-1, TNF-α, TGF-β, VEGF, MMP-1 and MMP-2 by transcriptional or post-transcriptional mechanisms. We conclude that vitamin D is beneficial to melanoma cells through the inhibition of oxidative DNA/RNA damage, membrane damage, and the expression of inflammatory, angiogenic and ECM remodeling proteins; and the stimulation of superoxide dismutase expression and p53 promoter activity.

## 1. Introduction

Carcinogenesis is associated with oxidative stress, inflammation, angiogenesis, and metastasis. The oxidative stress is from increased reactive oxygen species (ROS), which include hydroxyl radicals, superoxide, and hydrogen peroxide; and reduced counteracting antioxidants, which include superoxide dismutase, catalase, glutathione peroxidase, glutathione, ascorbate, α-tocopherol, and carotene. The ROS attack the DNA, proteins, and lipids directly. The oxidative damage to DNA and RNA includes the formation of 8-oxo-7, 8-dihydro-2′-deoxyguanosine (8-oxo-dG) or 8-oxo-7, 8-dihydro-2′-guanosine (8-oxo-G), which are increased in cancers [1,2,3,4,5,6,7,8,9,10,11,12]. The superoxide dismutases (SOD), including the mitochondrial MnSOD, are associated with gene mutations or altered expressions in cancers [4,6]. The ROS induce the generation of plasma mediators, lipid mediators, and the inflammatory cytokines such as interleukin-1 (IL-1), and tumor necrosis factor-α (TNF-α) [9,10,11,12,13,14,15,16,17,18,19]. The IL-1 and TNF-α, along with interleukin-6 (IL-6), are the three cytokines released by the activated tissue macrophages [13]. The inflammatory cytokines activate the mitogen activated protein kinase (MAPK), the nuclear factor-kappa beta (NF-kB)/p65, and the JAK/STAT (Signal Transduction and Activation of Transcription) pathways, which in turn induce the expression of angiogenic mediators such as transforming growth factor-β (TGF-β), and vascular endothelial growth factor (VEGF), and the ECM remodeling proteins such as the matrix metalloproteinases (MMP) [9,10,11,12,13,14,15,16,17,18,19,20]. While MMP-1 largely degrades the interstitial collagen, MMP-2 degrades the basement membrane.

Phenolic compounds, plant extracts, vitamins, and hormones with antioxidant and anti-inflammatory properties inhibit cellular oxidative stress, and the inflammatory, angiogenic and ECM remodeling proteins, and are anti-carcinogenesis or anti-aging [1,2,3,4,5,6,7,8,9,10,11,12,21,22,23,24,25,26,27,28,29]. The structure of 1α,25-dihydroxyvitamin D3 (vitamin D) and its endocrine, antioxidant, and anti-inflammatory properties lend to anti-carcinogenesis [29,30,31,32,33,34,35,36,37,38,39,40,41,42,43,44,45]. The epidemiological studies have not yet conclusively determined the anti-carcinogenic role of vitamin D [33]. However, the deficiency of vitamin D is linked to increased risk of cancer [33]. Vitamin D inhibits oxidative DNA damage in keratinocytes and non-irradiated or ultraviolet (UV) radiated fibroblasts, inhibits membrane damage in UV radiated fibroblasts, and inhibits lactate dehydrogenase levels that is indicative of tissue damage in response to exhaustive exercise, indicating protection from oxidative stress [29,34,35]. The exposure of skin to UV radiation is the key to the etiology of malignant melanoma, which arises from the melanocytes in the basal layer of the epidermis. There is co-ordinate regulation of melanoma cells and dermal fibroblasts by actives with antioxidant and/or anti-inflammatory properties, such as lutein, *P. leucotomos* extract, and *H. lupulus* extract [22,26,28]. The vitamin D receptor knock out mice exhibit reduced p53 levels, and premature aging [36]. UV radiation, directly and through ROS, reduces the expression or activity of the tumor suppressor p53, which causes cells to resist apoptosis and/or DNA repair [7,20]. Vitamin D regulates innate and adaptive immunity [37]. Its deficiency is associated with increased serum levels of TNF-α in asthma patients, as well as several other inflammatory diseases [38,39]. Vitamin D inhibits IL-1 expression in psoriasis as well as in non-irradiated or UV radiated fibroblasts; and TNF-α, through the inhibition of NF-kB activity, in peritoneal macrophages [29,40,41]. It also inhibits angiogenesis in vitro, in vivo, and in azoxymethane-induced colon carcinogenesis; as well as the expression of MMPs in human lung fibroblasts, and uterine fibroid cells [42,43,44,45].

In summary, carcinogenesis is associated with increased cell growth, angiogenesis, and metastasis, from the remodeling of the ECM, which are potentiated by the oxidative stress and inflammation that are induced by UV radiation and environmental pollutants. The 1α,25-dihydroxyvitamin D3 (vitamin D) exhibits direct antioxidant activity, and anti-inflammatory effects in non-irradiated and UV radiated fibroblasts [29]. Hence, the hypothesis of this research was that the structure of 1α,25-dihydroxyvitamin D3 (vitamin D), and its endocrine, anti-oxidant, and anti-inflammatory properties would lend to its beneficial regulation of cellular oxidative stress effects (oxidative DNA/RNA damage, SOD expression, membrane damage, and p53 promoter activity), and the expression (at the protein, mRNA and/or promoter levels) of inflammatory mediators (IL-1, and TNF-α), angiogenic mediators (TGF-β), and VEGF), and the ECM remodeling proteins (MMP-1 and MMP-2) by vitamin D in melanoma cells.

## 2. Results

### 2.1. Effect of 1α,25-Dihydroxyvitamin D3 (Vitamin D) on Oxidative DNA Damage, and Superoxide Dismutase Expression in Melanoma Cells

**Figure 1:**
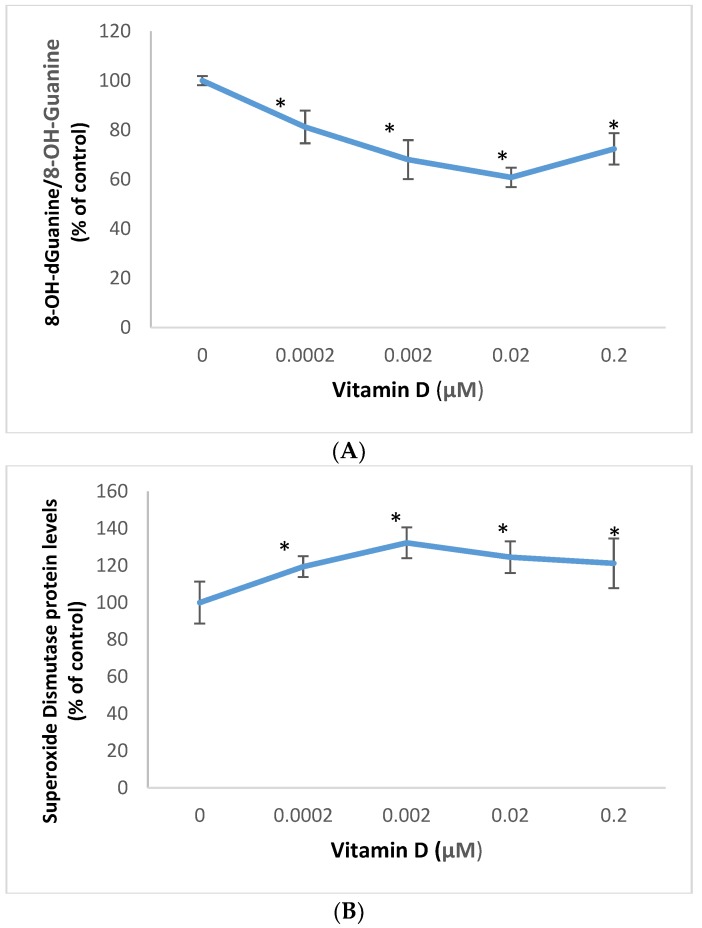
Effect of 1α,25-dihydroxyvitamin D3 (vitamin D) on 8-OH-dGuanine/8-OH-Guanine (oxidative DNA/RNA damage) (**A**), and superoxide dismutase protein levels (**B**) in melanoma cells; error bars: standard deviation, *n* = 4; * = *p* < 0.05, relative to control.

Vitamin D significantly inhibited oxidative DNA/RNA damage, and stimulated superoxide dismutase protein levels in melanoma cells (Figure 1).

Relative to the control (100%), vitamin D at 0.0002, 0.002, 0.02, and 0.2 μM significantly inhibited oxidative DNA/RNA damage to 81%, 68%, 61%, and 72% (*p* < 0.05) (Figure 1A); and significantly stimulated superoxide dismutase protein levels to 119%,132%,125%, and 121% (*p* < 0.05) (Figure 1B), in melanoma cells.

### 2.2. Effect of 1α,25-Dihydroxyvitamin D3 (vitamin D) on p53 Promoter Activity and Membrane Damage in Melanoma Cells

Vitamin D significantly stimulated p53 promoter activity, and inhibited membrane damage in melanoma cells (Figure 1).

**Figure 2:**
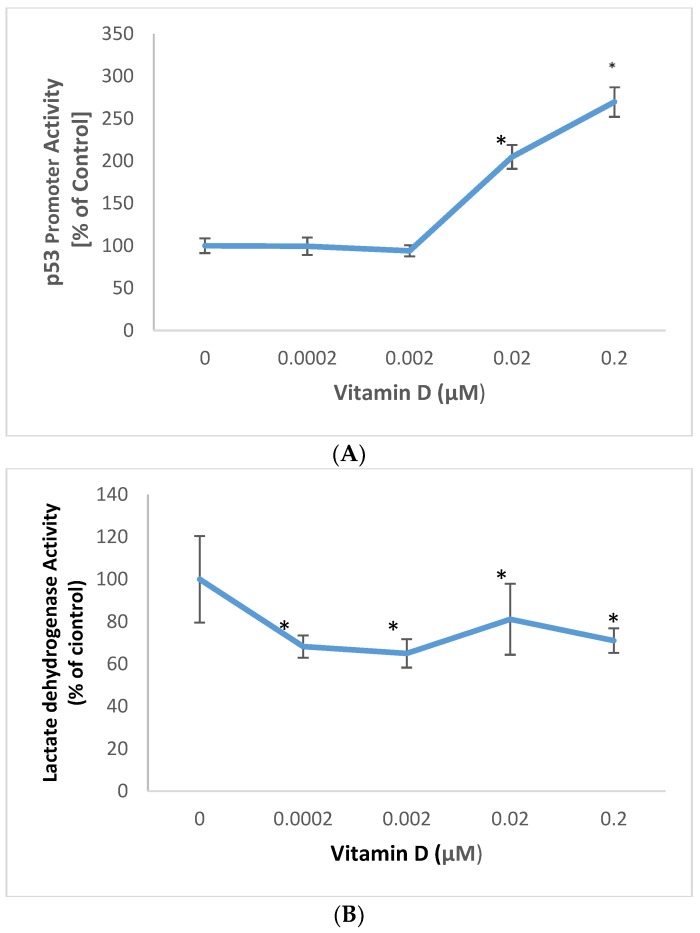
Effect of 1α,25-dihydroxyvitamin D3 (vitamin D) on p53 promoter activity (**A**), and lactate dehydrogenase activity (membrane damage) (**B**) in melanoma cells; error bars: standard deviation, *n* = 4; * = *p* < 0.05, relative to control.

Vitamin D at 0.02, and 0.2 µM significantly stimulated p53 promoter activity to 205%, and 270% of control (100%) in melanoma cells (*p* < 0.05) (Figure 2A). Relative to the control (100%), vitamin D at 0.0002, 0.002, 0.02, and 0.2uM significantly inhibited membrane damage to 68%, 65%, 81%, and 71% of control, in melanoma cells (*p* < 0.05) (Figure 2B)

### 2.3. Effect of 1α,25-Dihydroxyvitamin D3 (Vitamin D) on Interleukin-1 (IL-1) and Tumor Necrosis Factor Alpha (TNF-α) in Melanoma Cells

**Figure 3:**
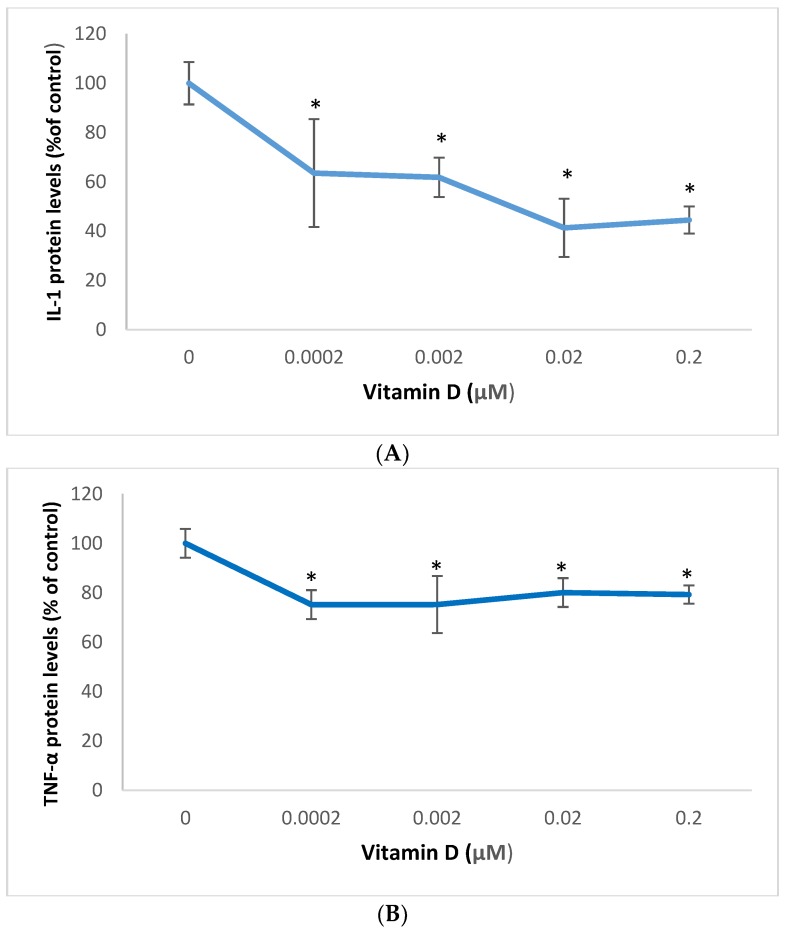
Effect of 1α,25-dihydroxyvitamin D3 (vitamin D) on interleukin-1 (IL-1) (**A**), and tumor necrosis factor alpha (TNF-α) (**B**) protein levels in melanoma cells; error bars: standard deviation, *n* = 4; * = *p* < 0.05, relative to control.

**Figure 4:**
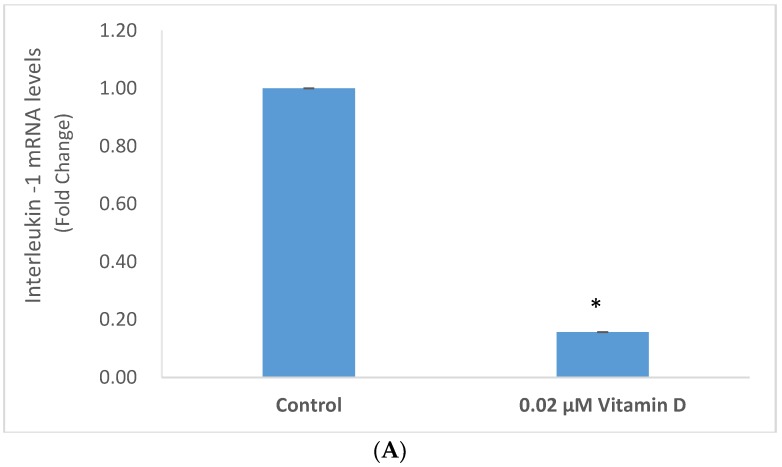

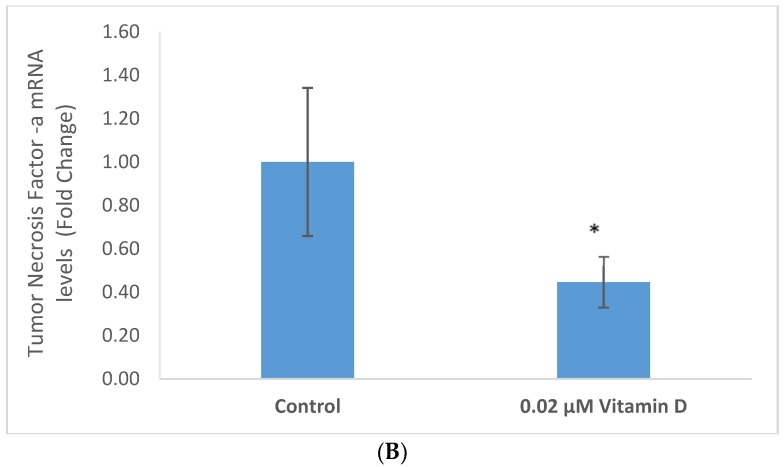
Effect of 0.02 µM 1α,25-dihydroxyvitamin D3 (vitamin D) on interleukin-1 (IL-1) (**A**), and tumor necrosis factor alpha (TNF-α) (**B**) mRNA levels in melanoma cells; error bars: standard deviation, *n* = 4; * = *p* < 0.05, relative to control.

Vitamin D significantly inhibited the expression of IL-1 and TNF-α in melanoma cells (Figure 3 and Figure 4).

Relative to the control (100%), vitamin D at 0.0002, 0.002, 0.02, and 0.2 µM significantly inhibited IL-1 protein levels to 63%, 62%, 41%, and 44% of control (*p* < 0.05) (Figure 3A); and significantly inhibited TNF-α protein levels to 75%, 75%, 80%, and 79% of control (*p* < 0.05) (Figure 3A), in melanoma cells.

Vitamin D at 0.02 µM significantly inhibited the mRNA levels of IL-1 and TNF-α to 0.16 and 0.45 fold, respectively, of control (*p* < 0.05) (Figure 4A,B).

### 2.4. Effect of 1α,25-Dihydroxyvitamin D3 (vitamin D) on Transforming Growth Factor Beta (TGF-β), and Vascular Endothelial Growth Factor (VEGF) in Melanoma Cells

**Figure 5:**
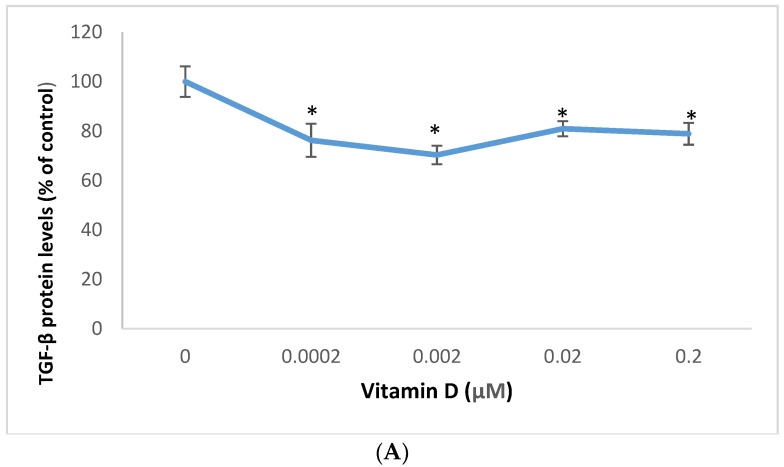

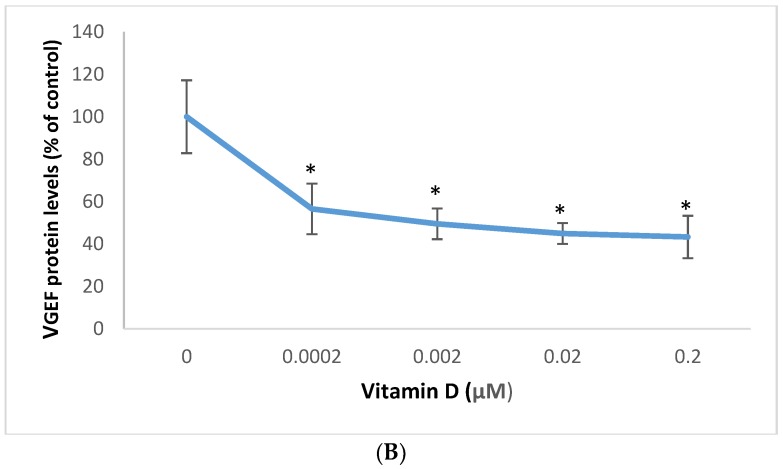
Effect of 1α,25-dihydroxyvitamin D3 (vitamin D) on transforming growth factor beta (TGF-β) (**A**), and vascular endothelial growth factor (VEGF) (**B**) protein levels in melanoma cells; error bars: standard deviation, *n* = 4; * = *p* < 0.05, relative to control.

**Figure 6:**
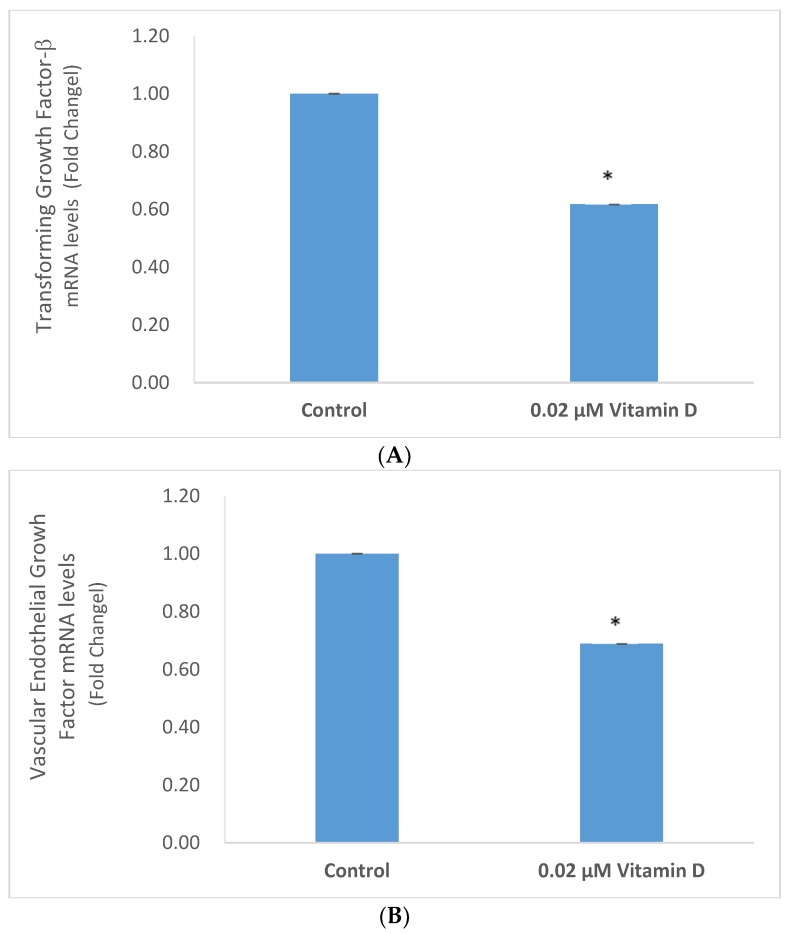
Effect of 0.02 µM 1α,25-dihydroxyvitamin D3 (vitamin D) on transforming growth factor beta (TGF-β) (**A**), and vascular endothelial growth factor (VEGF) (**B**) mRNA levels in melanoma cells; error bars: standard deviation, *n* = 4; * = *p* < 0.05, relative to control.

Vitamin D significantly inhibited the expression of TGF-β and VEGF in melanoma cells (Figure 5 and Figure 6).

Relative to the control (100%), vitamin D at 0.0002, 0.002, 0.02, and 0.2 µM significantly inhibited TGF-β protein levels to 76%, 70%, 81%, and 79% of control (*p* < 0.05) (Figure 5A); and significantly inhibited VEGF protein levels to 57%, 49%, 45%, and 43% of control (*p* < 0.05) (Figure 5B), in melanoma cells.

Vitamin D at 0.02 µM significantly inhibited the mRNA levels of TGF-β and VEGF to 0.62 and 0.69 fold, respectively, of control (*p* < 0.05) (Figure 6A,B).

### 2.5. Effect of 1α,25=Dihydroxyvitamin D3 (vitamin D) on Matrixmetalloproteinase (MMP)-1 and MMP-2 in Melanoma Cells

**Figure 7:**
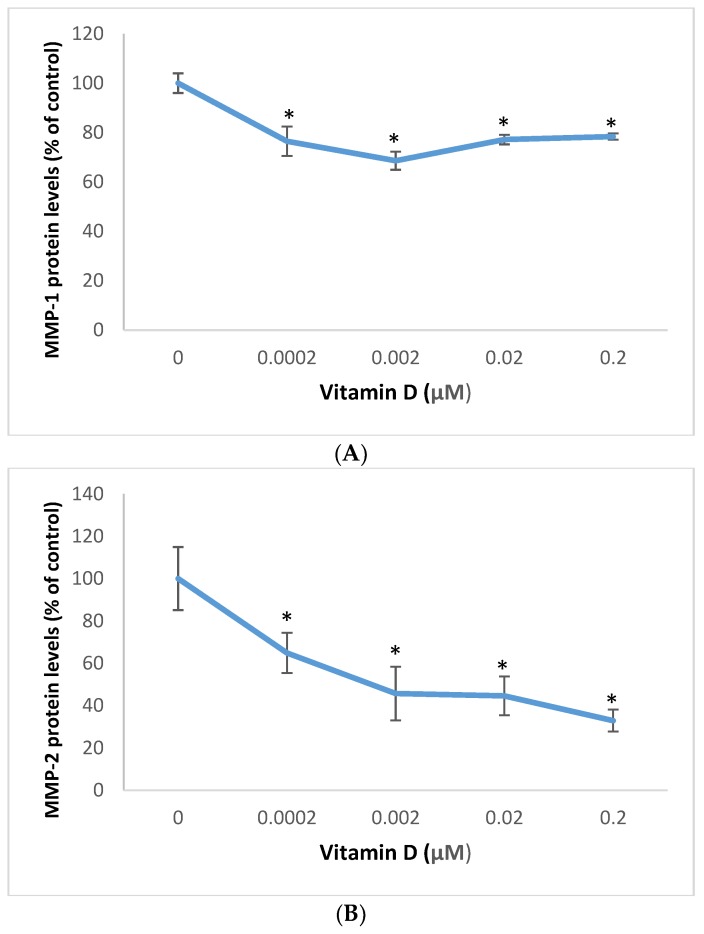
Effect of 1α,25-dihydroxyvitamin D3 (vitamin D) on Matrix metalloproteinase (MMP)-1 (**A**), and MMP-2 (**B**) protein levels in melanoma cells; error bars: standard deviation, *n* = 4; * = *p* < 0.05, relative to control.

Vitamin D significantly inhibited MMP-1 and MMP-1 protein levels in melanoma cells (Figure 7). It did not significantly inhibit the MMP-1 promoter activity, or the MMP-1 or MMP-2 mRNA levels in these cells (data not shown).

Relative to the control (100%), vitamin D at 0.0002, 0.002, 0.02, and 0.2 µM significantly inhibited MMP-1 protein levels to 76%, 69%,77%, and 78% of control (*p* < 0.05) (Figure 7A); and significantly inhibited MMP-2 protein levels to 65%, 46%,45%, and 33% of control (*p* < 0.05) (Figure 7A), in melanoma cells.

## 3. Discussion

Carcinogenesis is associated with increased oxidative stress, inflammation, angiogenesis, and ECM remodeling. The alterations collectively enable the cancer cells to grow and metastasize. We have reported the beneficial regulation of cellular and extracellular parameters by plant extracts, phenolic compounds, hormones, and vitamins in the amelioration of aging, photoaging or photocarcinogenesis [21,22,23,24,25,26,27,28,29,46,47,48,49,50,51,52]. We recently reported the inhibition of cellular oxidative stress effects and inflammation by vitamin D in non-irradiated, and UV radiated fibroblasts [29]. Vitamin D had been reported to inhibit oxidative stress, inflammation, angiogenesis and ECM remodeling in diverse cell types or tissue [30,31,32,33,34,35,36,37,38,39,40,41,42,43,44,45]. We herein extend the beneficial effects of vitamin D to melanoma cells.

The cellular oxidative stress as well as the reduced expression of antioxidant enzymes and tumor suppressor p53 occurs with intrinsic aging, and additionally with exposure to environmental pollutants and UV radiation. Vitamin D significantly inhibited oxidative DNA/RNA damage, and membrane damage; and stimulated superoxide dismutase protein levels, and p53 promoter activity in melanoma cells. It is inferred that vitamin D is effective in directly inhibiting oxidative stress effects and inducing p53 expression that facilitates cell cycle arrest or apoptosis [20].

Several cell types release the inflammatory mediators, and the initial inflammatory response is through innate or non-specific immunity [13,14,15]. The key inflammatory cytokines, IL-1 and TNF-α, activate the MAPK, JAK/STAT and NF-kB pathways, which in turn activate transcription factors to induce further inflammatory, angiogenic, and ECM modulating factors [10,11,12,13,14,15,16,17,18,19,20]. Vitamin D inhibited IL-1 and TNF-α protein and mRNA levels, suggesting transcriptional mechanism; and its effectiveness in inhibiting inflammation in melanoma cells.

The key factors that regulate angiogenesis and metastasis are TGF-β, VEGF, and MMPs. TGF-β regulates the cell cycle, angiogenesis, and the extracellular matrix; and has differential effects in different cell types [9,10,11,12,20]. VEGF is key to angiogenesis, and the MMPs to the degradation and remodeling of the ECM. There is coordinate regulation of TGF-β, VEGF, and/or MMPs in cancer cells [9,10,11,12,20,50,52,53]. Vitamin D inhibited TGF-β, and VEGF at the protein and mRNA levels, suggesting transcriptional mechanism, and MMP-1, and MMP-2 at the proteins level, suggesting post-transcriptional mechanism; and its effectiveness in inhibiting angiogenic and metastatic potential in melanoma cells.

Overall, it is concluded that vitamin D is beneficial to melanoma cells through the inhibition of oxidative DNA/RNA damage, membrane damage, and the expression of inflammatory, angiogenic and ECM remodeling proteins; and the stimulation of superoxide dismutase expression and p53 promoter activity. The Vitamin D concentrations of 0.0002 µM, 0.002 µM, 0.02 µM, and 0.2 were effective in significantly regulating all targets examined in a melanoma cell line, except for p53 promoter activity that was upregulated by Vitamin D concentrations of 0.02 µM and 0.2 µM.

## 4. Method

### 4.1. Cell Culture/Experimental Design

Melanoma cells (CRL-1619, American Tissue Cell Culture) were cultured in Dulbecco’s Modified Eagle’s Medium (DMEM) supplemented with 10% heat inactivated fetal bovine serum and 1% penicillin/streptomycin (Life Technologies, Sigma) [22,26,28,46,47]. Melanoma cells were incubated with 0, 0.0002, 0.002, 0.02 or 0.2 µM 1α,25–dihydroxyvitamin D3 (D1530, Sigma, dissolved in DMSO to a 20 mM stock solution) in DMEM supplemented with 3% heat inactivated fetal bovine serum for 24 h for experiments, to measure cell viability, oxidative DNA/RNA damage, membrane damage, and expression of p53, superoxide dismutase (SOD), interleukin-1 (IL-1), tumor necrosis factor-α (TNF-α), transforming growth factor-β (TGF-β), vascular endothelial growth factor (VEGF), matrix metalloproteinases (MMP)-1 and MMP-2 at the protein and/or promoter levels. The cells were incubated with 0 or 0.02 uM vitamin D to measure the expression of IL-1, TNF-α, TGF-β, VEGF, MMP-1, and MMP-2 at the mRNA levels

### 4.2. Cell Viability

Vitamin D at 0.0002–0.02uM did not alter the viability of melanoma cells, relative to control (data not shown). The cells were examined for cell viability by the CellTiter 96^®^ Aqueous One Solution reagent (tetrazolium compound (3-(4,5-dimethylthiazol-2-yl)-5-(3-carboxymethoxyphenyl)-2-(4-sulfophenyl)-2H-tetrazolium, inner salt; MTS) and electron coupling reagent (phenazine ethosulfate; PES)] (Promega); by incubating the cells, after the 24 h of incubation with vitamin D, with aliquots of the MTS reagent (yellow) for 30 min at 37 °C, and measuring the product (brown, through the conversion of the tertrazolium reagent to formazan by viable cells) spectrophotometrically at 490 nm [21,22,23,24,25,26,27,28,29,46,47,48,49,50,51,52].

### 4.3. Oxidative DNA/RNA Damage

The oxidative DNA/RNA damage was measured with the competitive DNA/RNA Oxidative Damage ELISA Kit (Cayman Chemical) [29]. Aliquots of cells or buffer were incubated with competitive acetylcholinesterase linked to 8-OH-dG (tracer), and antibody to oxidatively damaged guanine for 24 h at 4 °C, washed, incubated with substrate, and the product measured spectrophotometrically at 412 nm. The readings were subtracted from the maximum tracer binding (buffer) to determine the cellular DNA/RNA oxidative damage.

### 4.4. Protein Levels: Total Protein Content and the Protein Levels of Superoxide Dismutase (SOD), Interleukin-1 (IL-1), Tumor Necrosis Factor-α (TNF-α), Transforming Growth Factor-β (TGF-β), Vascular Endothelial Growth Factor (VEGF), Matrix Metalloproteinases (MMP)-1, and MMP-2

Vitamin D at 0.0002–0.02 µM did not alter the total protein content (extracellularly or intracellularly) of melanoma cells, relative to control (data not shown). The total protein content in the media and cells, following experiments, was determined by the Pierce Bicinchoninic Acid (BCA) Protein Assay (Thermo Fischer Scientific, Waltham, MA, USA); by incubating aliquots with the BCA and cupric ion reagent, and measuring the formation of BCA-cuprous ion, proportional to the total protein content, spectrophotometrically at 562 nm.

The expression of SOD, IL-1, TNF-α, TGF-β, VEGF, MMP-1, and MMP-2 proteins was measured by indirect ELISA (Kirkguaard and Perry Laboratories, Inc) [21,22,23,24,25,26,27,28,29,46,47,48,49,50,51,52]. The aliquots of cells or media were incubated with coating buffer in 96-well immunosorbent plates for 24 h at 4 °C, blocked with bovine serum albumin for 30 min at room temperature, incubated with respective primary antibodies (Cayman Chemical, Millipore, Sigma, St.Louis, MO, USA) for 1 h at room temperature, washed with wash buffer, incubated with secondary antibody linked to peroxidase for 1 h at room temperature, washed, and incubated with peroxidase substrate until color development, and measured spectrophotometrically at 405 nm.

### 4.5. Promoter Activities: p53, and MMP-1

The melanoma cells were co-transfected with p53 promoter-firefly luciferase (pGL4 vector), plasmid (Promega) and TK-h Renilla luciferase plasmid (Promega), for normalization of transfection efficiency, with Escort (Sigma); prior to incubation with or without vitamin D [21,23,25,26,28,29,47,48]. The cells were measured for luminescence due to the firefly, and renilla luciferase activities sequentially with the Dual Luciferase Reporter Assay kit (Promega) [21,25,26,29,47].

Vitamin D at 0.0002-0.02uM did not significantly alter the MMP-1 promoter activity in melanoma cells, relative to control (data not shown). Melanoma cells were co-transfected with the MMP-1 promoter-chloramphenicol acetyl transferase (CAT) plasmid (gift from Dr. William C. Parks, Washington University School of Medicine, St. Louis, MO) and RSV2-β Galactosidase (β-GAL) (Sigma), for normalization of transfection efficiency, with Escort (Sigma); prior to incubation with or without vitamin D [21,23,25,26,28,29,47,48]. The cells were examined for CAT expression by ELISA, and for β-GAL activity by β-Galactosidase Enzyme Assay System (Promega) [23,28,48].

### 4.6. Membrane Damage

The media were examined for lactate dehydrogenase (LDH) activity, indicative of membrane damage, with the LDH toxicity kit (Sigma, Tox-7) [27,29]. The aliquots of media were incubated with the LDH substrate, cofactor and tertrazolium dye reagents, and the reduction of the tertrazolium dye was measured spectrophotometrically at 490nm.

### 4.7. RNA Levels: IL-1, TNF-α, TGF-β, VEGF, MMP-1 and MMP-2 mRNA, and 18S RNA

The RNA was extracted from cells, following incubation with or without vitamin D, using the RNeasy Plus Mini Kit (Qiagen) [49]. Vitamin D did not significantly alter the total RNA content, relative to control; which was measured by incubating aliquots of the extracted RNA with a fluorescent dye and measuring fluorescence at 490 nm excitation/540 nm emission (QuantiFluor RNA system, Promega, Madison, WI, USA). Vitamin D did not significantly alter the mRNA levels of MMPs, relative to control (data not shown). The aliquots of RNA were reverse transcribed to cDNA with the iScript cDNA Synthesis Kit (Bio-Rad, Hercules, CA, USA), and analyzed for IL-1, TNF-α, TGF-β, VEGF, MMP-1, MMP-2, and 18S (for normalization, as internal control) cDNA levels by quantitative-polymerase chain reaction (qPCR) using specific primers (Qiagen) and SsoAdvanced Universal SYBR Green Supermix (Bio-Rad). The fold change of mRNA expression was calculated by the 2^(-Delta Delta Ct)^ method (Qiagen).

### 4.8. Data Analysis

The data were analyzed for significant difference by ANOVA and student *t*-tests at 95% confidence interval.
